# Monoamine control of descending pain modulation after mild traumatic brain injury

**DOI:** 10.1038/s41598-022-20292-7

**Published:** 2022-09-29

**Authors:** Peyman Sahbaie, Karen-Amanda Irvine, Xiao-you Shi, J. David Clark

**Affiliations:** 1 Department of Anesthesiology, Perioperative and Pain Medicine, School of Medicine, Stanford University, Stanford, CA 94305 USA; 2 Anesthesiology Service, Veterans Affairs Palo Alto Health Care System, 3801 Miranda Ave (E4-220), Palo Alto, CA 94304 USA

**Keywords:** Neuroscience, Pain

## Abstract

Traumatic brain injury (TBI) is a significant public health concern, with the majority of injuries being mild. Many TBI victims experience chronic pain. Unfortunately, the mechanisms underlying pain after TBI are poorly understood. Here we examined the contribution of spinal monoamine signaling to dysfunctional descending pain modulation after TBI. For these studies we used a well-characterized concussive model of mild TBI. Measurements included mechanical allodynia, the efficacy of diffuse noxious inhibitory control (DNIC) endogenous pain control pathways and lumber norepinephrine and serotonin levels. We observed that DNIC is strongly reduced in both male and female mice after mild TBI for at least 12 weeks. In naïve mice, DNIC was mediated through α2 adrenoceptors, but sensitivity to α2 adrenoceptor agonists was reduced after TBI, and reboxetine failed to restore DNIC in these mice. The intrathecal injection of ondansetron showed that loss of DNIC was not due to excess serotonergic signaling through 5-HT_3_ receptors. On the other hand, the serotonin-norepinephrine reuptake inhibitor, duloxetine and the serotonin selective reuptake inhibitor escitalopram both effectively restored DNIC after TBI in both male and female mice. Therefore, enhancing serotonergic signaling as opposed to noradrenergic signaling alone may be an effective pain treatment strategy after TBI.

## Introduction

Traumatic brain injury (TBI) is a significant public health concern. In the US, more than 2.5 million TBIs are reported each year resulting in an estimated death rate of 17.3 per 100,000 population^1^. Common causes include falls, sports-related injuries, motor vehicle accidents, assaults and blast injuries. The majority of injuries are mild and involve short-term (< 30 min) loss of consciousness^2^. The types and patterns of disabilities after TBI have been well described and include motor and balance dysfunction, cognitive difficulties and emotional problems^3^. Pain, however, has been an often-overlooked consequence despite complicating rehabilitation efforts and constituting an ongoing management challenge in many patients^4^. More recently, the interaction of physical, emotional and pain-related problems has been recognized; chronic pain and post-traumatic stress disorder after TBI co-occur in the so-called polytrauma clinical triad^5^.

The prevalence, characteristics and potential mechanisms for chronic pain after TBI have been reviewed recently^6–8^. These analyses suggest that chronic pain persists after TBI in more than 60% of those sustaining such injuries and that pain incidence may, paradoxically, be higher amongst those with mild as opposed to more severe injuries. The most common type of both acute and chronic pain after TBI is headache pain, although pain experienced in the limbs, neck and back can occur^9,10^. Interestingly, examination of endogenous pain control systems including conditioned pain modulation (CPM) have suggested that these systems are disrupted in TBI patients^11^ and that such deficiencies in TBI patients predict chronic pain^12^. Imaging studies have suggested that TBI patients may sustain damage to endogenous pain control centers such as the periaqueductal gray matter (PAG)^13^.

Animal models continue to be useful approximations of human brain trauma and remain critical to our understanding of TBI and potential treatment modalities. Our lab and others have studied pain post-injury using various models of TBI that show mechanical sensitization in the periorbital region of the head, forelimbs and hindlimbs often transient over several days^14–20^. In a rat lateral fluid percussion model, dysfunction of endogenous pain modulation was demonstrated along with histopathological evidence of damage to or neuroinflammation of key endogenous pain control centers such as the LC, PAG and rostro-ventromedial medulla (RVM). These centers send fibers to the dorsal horn of the spinal cord to modulate nociceptive signaling^19^. Augmenting serotonin signaling via administration of reuptake inhibitors restored one type of endogenous pain modulation, “diffuse noxious inhibitory control” or “DNIC,” the animal testing counterpart to CPM, in post-TBI rats more than 6 months from the time of their injuries^21^. While these foundational observations are important, the most common type of TBI, concussion, does not involve penetrating injury to the brain^22^. In these studies we set out to expand our understanding of pain after brain injury using a model of concussive TBI, studying TBI in an additional species, more closely examining changes in spinal monoamine signaling, and extending key observations to female mice.

## Results

### Long term failure of diffuse noxious inhibitory control (DNIC) response in mice after TBI

**Figure 1:**
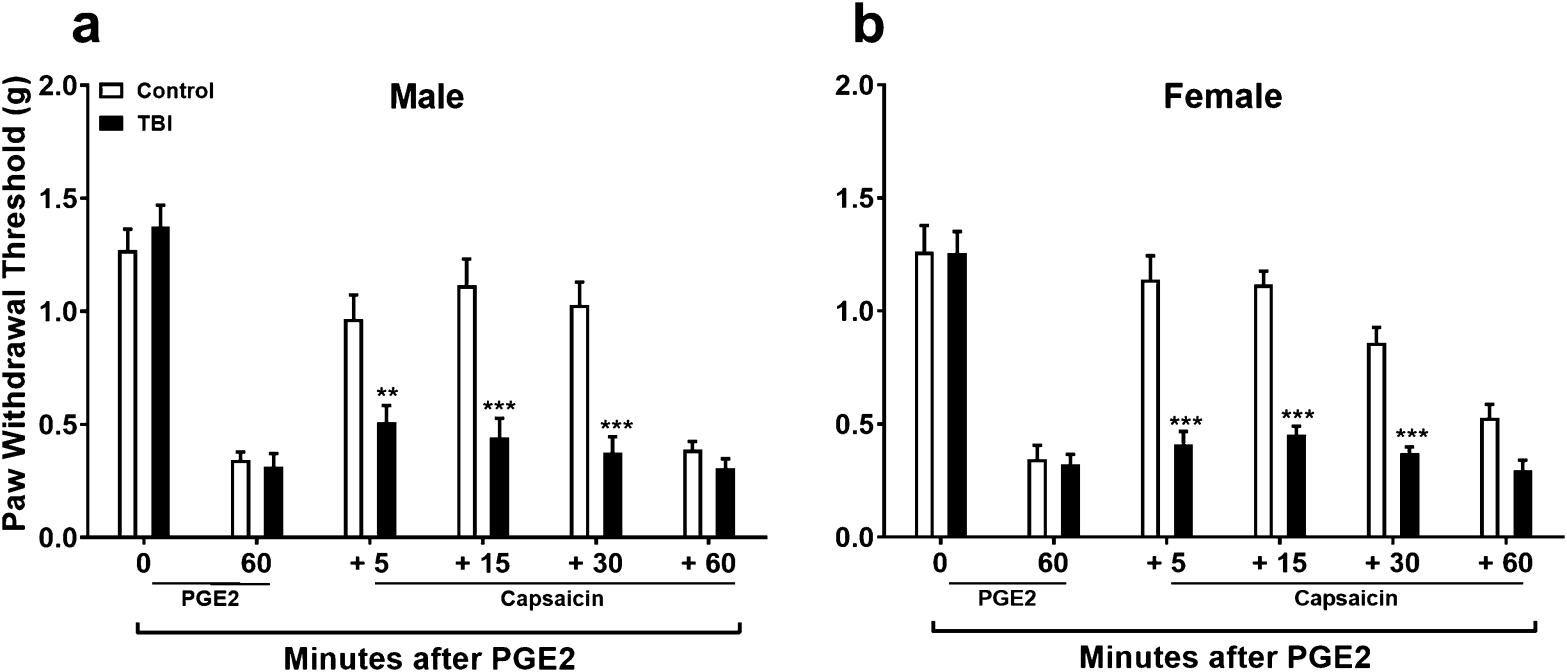
Long term assessment of diffuse noxious inhibitory control (DNIC) after mild TBI. Results show that the DNIC deficit remains impaired in both male (**a**) and female (**b**) mice at 12 weeks post-TBI. Data were analyzed by two-way repeated measures ANOVA using the Sidak method to correct for multiple comparisons. **p < 0.01, ***p < 0.001 indicates significant difference between sham control and TBI. Error bars: SEM, n = 6/group.

We have previously shown that following a closed-head injury, mice undergo an acute period of nociceptive sensitization of the hindpaws that peaks at 72 h. This period is resolved within 14–21 days but DNIC declines within 4 weeks of injury^19^. In this study we expanded our observations by conducting DNIC testing at 12 weeks post closed head TBI to determine if loss of endogenous pain modulation was present in mice at later time points. Results showed that the DNIC deficit remained impaired in both male and female mice at 12 weeks post-TBI (Fig. 1). Between group analysis revealed no significant male vs. female DNIC response differences for sham (F_1,10_ = 0.06, *p* = 0.81) or TBI (F_1,10_ = 0.36, *p* = 0.57) procedures.

### The role of norepinephrine signaling in diffuse noxious inhibitory control (DNIC) response

**Figure 2:**
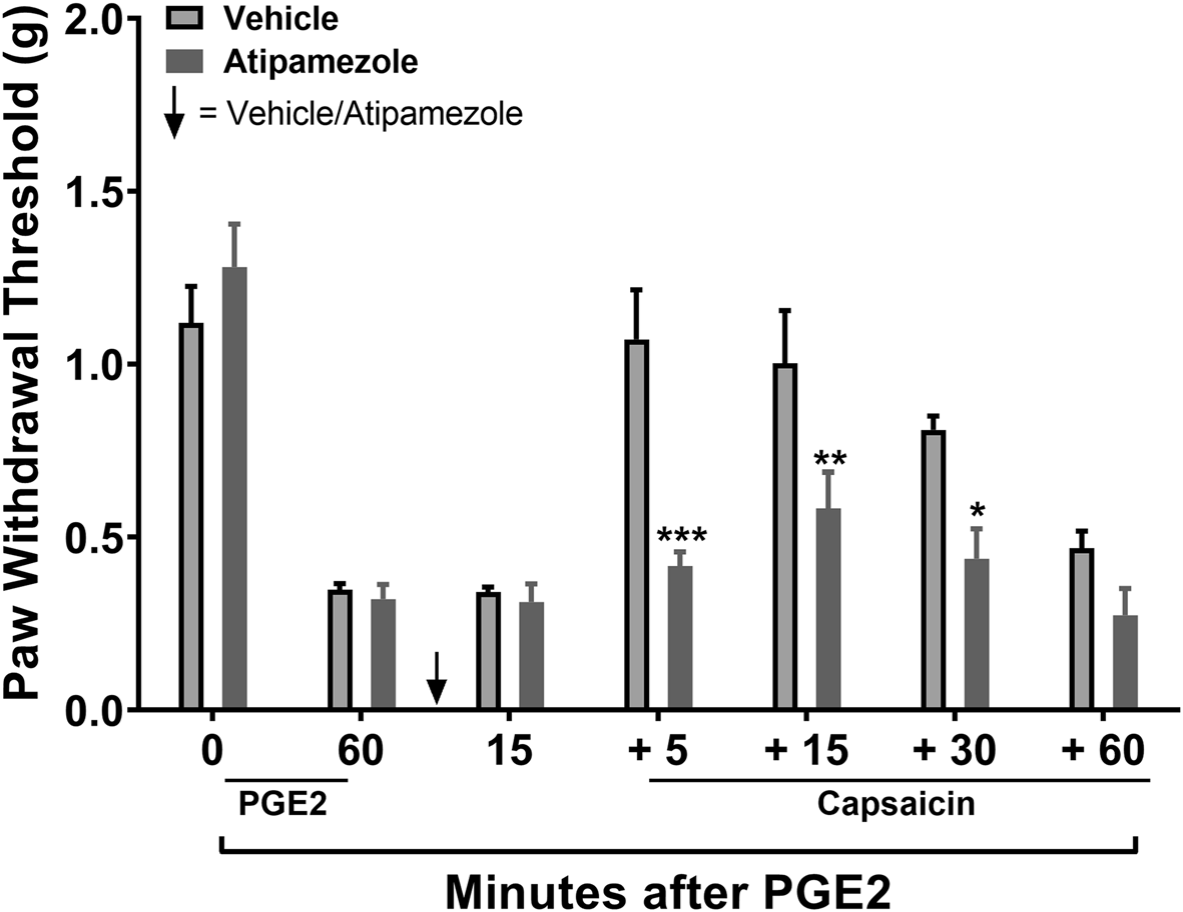
Assessment the role of norepinephrine signaling in diffuse noxious inhibitory control (DNIC) in naive mice. The α2 adrenoceptor antagonist, atipamezole (ATZ) given systemically prior to injection of the conditioning stimulus, capsaicin, in naïve male mice had no effect on the PGE-2-induced sensitization. However, ATZ did block the DNIC response to capsaicin in non-injured mice. Data were analyzed by two-way repeated measures ANOVA using the Sidak method to correct for multiple comparisons. *p < 0.05, **p < 0.01, ***p < 0.001 indicates significant difference between vehicle and drug treatment groups. Error bars: SEM, n = 6/group.

We and others have shown that norepinephrine (NE) signaling plays a key role in the DNIC response in naïve rats. To verify its role in another species, mice, we used the α2 adrenoceptor antagonist, atipamezole (ATZ) prior to injection of the conditioning stimulus, capsaicin, in naïve mice. Behavioral testing 15 min after ATZ injection revealed that it had no effect on the PGE_2_ induced sensitization. However, ATZ did block the DNIC response to capsaicin in non-injured mice demonstrating an important role for spinal adrenergic input to regulate descending pain control under basal conditions (Fig. 2: F_1,10_ = 8.43, *p* < 0.05 ; significant main effect of treatment).

### Systemic treatment with the norepinephrine reuptake inhibitor, reboxetine, fails to restore the diffuse noxious inhibitory control (DNIC) response in TBI mice

**Figure 3:**
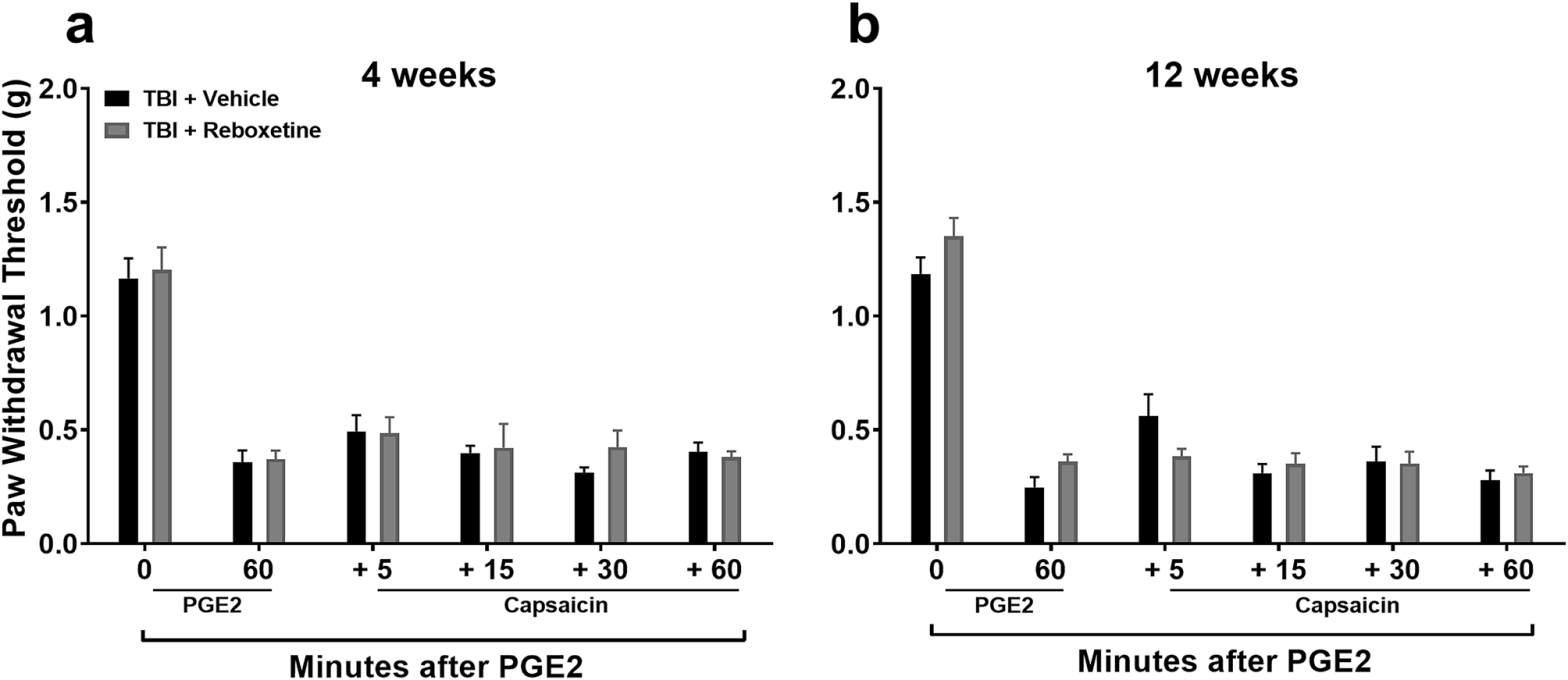
Assessment of diffuse noxious inhibitory control (DNIC) response after reboxetine treatment. Systemic pre-treatment with the norepinephrine reuptake inhibitor, reboxetine (RBX, 10 mg/kg, i.p. for 3 days) fails to restore the DNIC response at either 4 (**a**) or 12 (**b**) weeks post TBI in male mice. The last dose of RBX was given 18 h prior to testing to avoid acute analgesic effects of RBX on PGE_2_ application. Data were analyzed by two-way repeated measures ANOVA using the Sidak multiple comparisons test to correct for multiple comparisons. Error bars: SEM, n = 6/group.

In an attempt to restore the DNIC response in mice after TBI we aimed to augment the NE mediated descending inhibitory pathway by increasing its spinal levels using the NE reuptake inhibitor, Reboxetine (RBX). Pretreatment with RBX three days prior to DNIC testing was used to increase central NE^23^, the last dose was given 18 h prior to testing to avoid acute analgesic effects of RBX on PGE_2_ application. Results revealed that augmenting central NE levels failed to reinstate the DNIC response (Fig. 3) at both 4 (3a: F_1,10_ = 0.59, *p* = 0.46 ; no significant main effect of treatment) and 12 (3b: F_1,10_ = 0.35, *p* = 0.57 ; no significant main effect of treatment) weeks post TBI.

### Measurement of spinal levels of the key monoamine pain-related neurotransmitters after TBI

**Figure 4:**
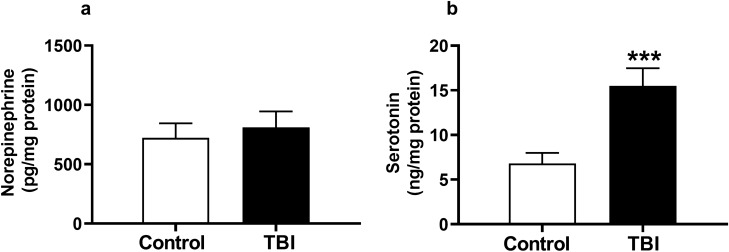
Assessment of spinal levels of the key monoamine pain-related neurotransmitters after mild TBI. Spinal NE and 5-HT levels 4 weeks after TBI at which time the DNIC response had failed were assessed. No significant difference in spinal lumbar NE leves were seen between male TBI and sham mice (**a**). In contrast, there was a significant increase in spinal 5-HT protein levels in TBI compared to control group (**b**). Data were analyzed by two tailed t tests for each protein levels comparision. Error bars: SEM, n = 8/group. *p* < 0.001 for comparison to control group.

The monoamines NE and serotonin (5-HT) are key spinal neurotransmitters participating in endogenous pain control systems. We therefore measured spinal NE and 5-HT levels 4 weeks after TBI at which time the DNIC response had failed. As hindpaw responses were evaluated throughout this work, lumbar spinal cord tissue was harvested 4 weeks post-TBI. No significant difference in the levels of spinal NE between male TBI and sham mice (Fig. 4a, *p* = 0.19) was observed. In contrast, there was a significant increase in spinal 5-HT protein levels between TBI and sham mice, the other key modulator of descending endogenous pain control circuits (Fig. 4b, *p* < 0.001).

### Lumbar spinal intrathecal administration of the α2 adrenoceptor agonist dexmedetomidine has a reduced analgesic response in TBI mice

**Figure 5:**
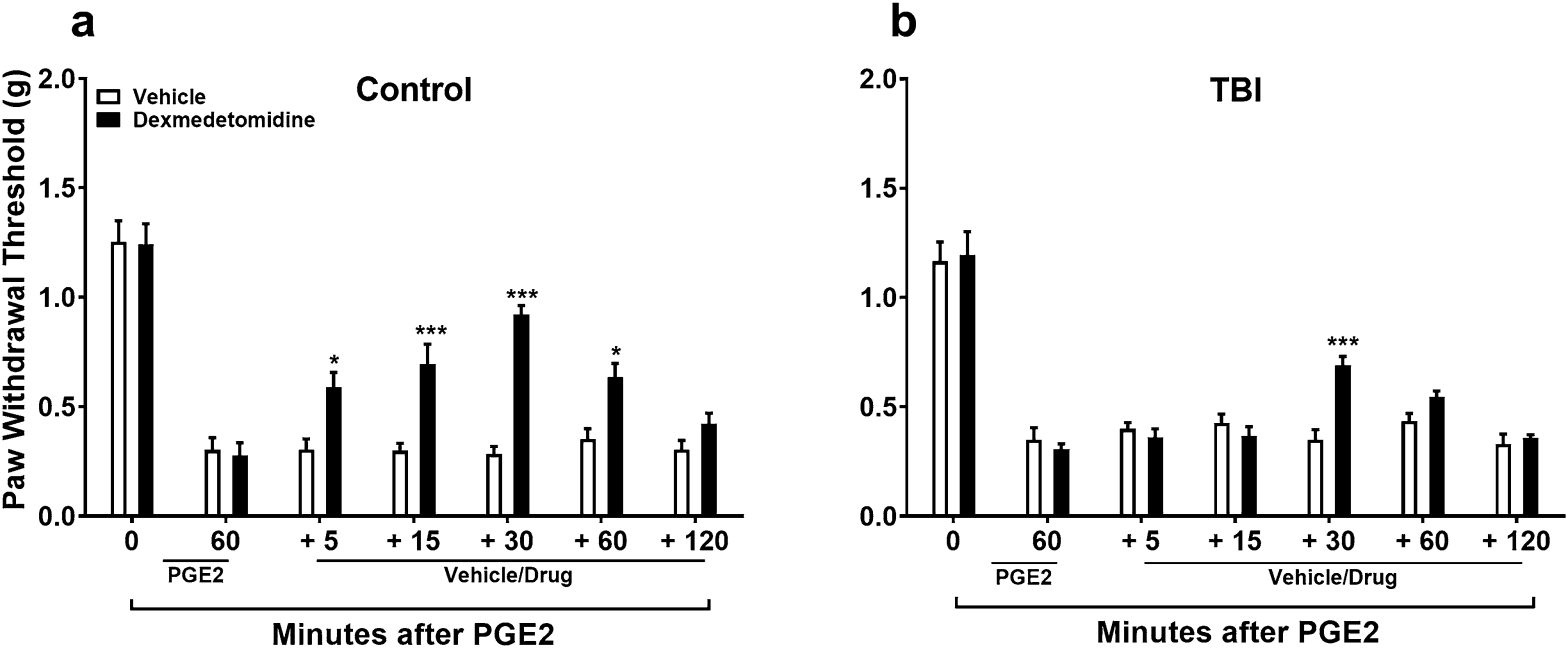
Assessment of norepinephrine signaling and analgesic response after mild TBI. Lumbar spinal intrathecal administration of the α2 adrenoceptor agonist dexmedetomidine (DEX) produced significant analgesia in control naïve male mice (**a**) when given after plantar PGE_2_-induced sensetization. DEX treatment had markedly reduced analgesic response to PGE_2_-induced sensetization when given 4 weeks after mild TBI (**b**). Data were analyzed by two-way repeated measures ANOVA using the Sidak method to correct for multiple comparisons. *p < 0.05, **p < 0.01, ***p < 0.001 indicates significant difference between vehicle and drug treatment groups. Error bars: SEM, n = 6/group.

A possible reason for the failure of RBX to restore the DNIC response in TBI mice is that the function of the spinal α2 adrenoceptors may be reduced. Therefore we proceeded with analgesic response evaluations after mild TBI. After initial hindpaw mechanical sensitization with local PGE_2_ application was achieved, the α2 adrenoceptor agonist dexmedetomidine (DEX) was given intrathecally in control and TBI groups (Fig. 5). Both groups were evaluated for 120 min post DEX application. DEX produced significant analgesia compared to vehicle treatment in control groups (5a: F_1,10_ = 32.71, *p* < 0.001 ; significant main effect of treatment). Results revealed mixed analgesic response to DEX when given 4 weeks post mild TBI (5b: F_6,60_ = 3.72, *p* < 0.01; significant treatment X time interaction). Post hoc Sidak multiple comparisons test showed significant statistical difference at the 30 min timepoint between vehicle and DEX groups (*p* < 0.001), while other timepoints were not significantly different between the two treatment groups.

### Reducing pronociceptive serotonergic signaling with the 5-HT_3_ receptor antagonist, ondansetron, fails to restore the diffuse noxious inhibitory control (DNIC) response in TBI mice

**Figure 6:**
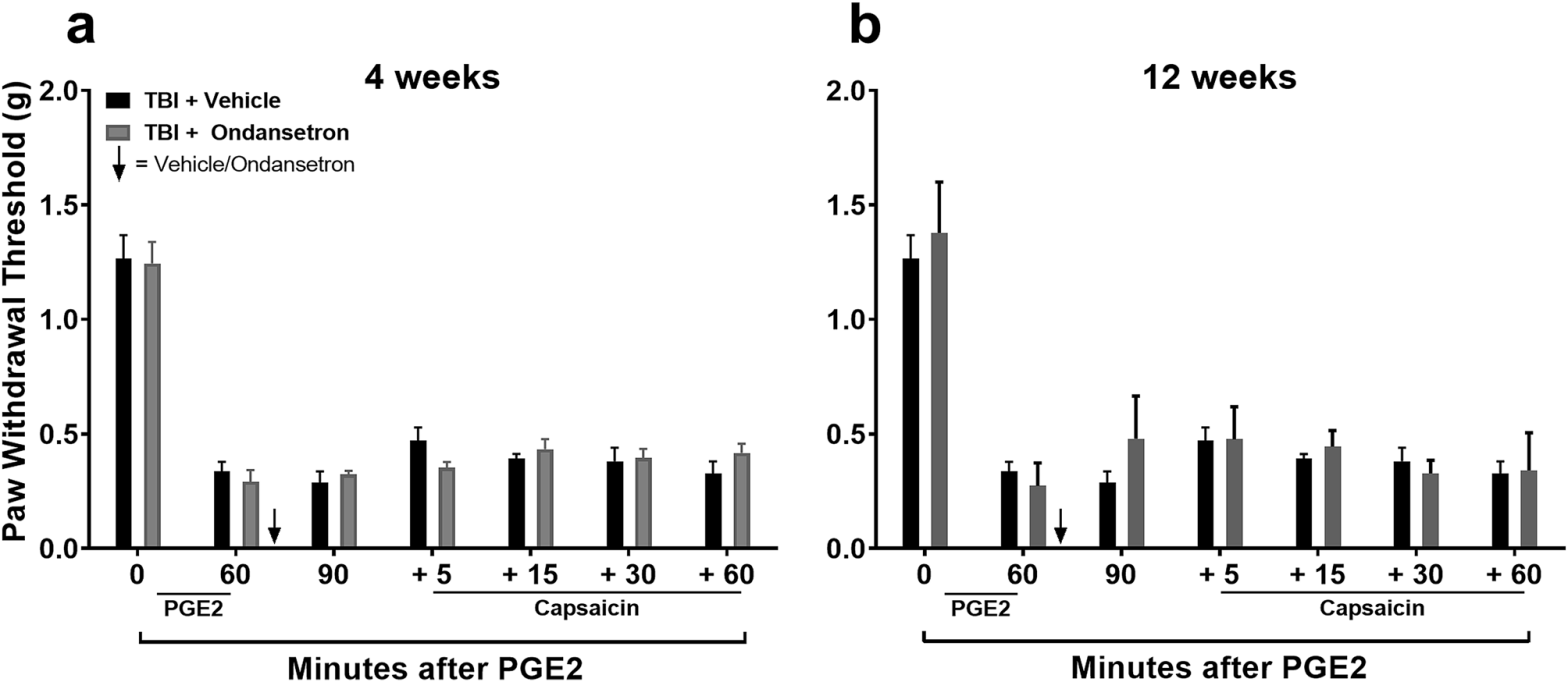
Effects of 5-HT_3_ receptor antagonist ondansetron on diffuse noxious inhibitory control (DNIC) response after mild TBI. The HT_3_ receptor antagonist, ondansetron (OND) was used to reduce spinal pronociceptive serotonergic signaling. Intrathecal OND fails to restore the DNIC response at 4 (**a**) or 12 (**b**) weeks post-TBI in male mice. Data were analyzed by two-way repeated measures ANOVA using the Sidak method to correct for multiple comparisons. Error bars: SEM, n = 6/group.

Aside from dysfunctional spinal NE signaling, DNIC failure in mice after TBI could be the imbalance between descending pain inhibition and descending pain facilitation mediated through spinal 5-HT receptors as shown to occur in models of neuropathic pain^24^. To investigate this possibility, mice were treated with intrathecal selective 5-HT_3_ receptor antagonist ondansetron (OND) after PGE_2_ but prior to capsaicin application in DNIC assessment. Results revealed that OND failed to restore the DNIC response (Fig. 6) at 4 (6a: F_1,10_ = 0.00, *p* = 0.99; no significant main effect of treatment) or 12 (6b: F_1,10_ = 0.86, *p* = 0.38; no significant main effect of treatment) weeks post-TBI. In addition, intra-thecal OND had no effect on mechanical sensitivity or the DNIC response of control/sham group (Figure S2).

### Diffuse noxious inhibitory control (DNIC) response was restored in TBI mice at 4- and 12-weeks post-TBI after systemic pretreatment with the serotonin and norepinephrine reuptake inhibitor, duloxetine

**Figure 7:**
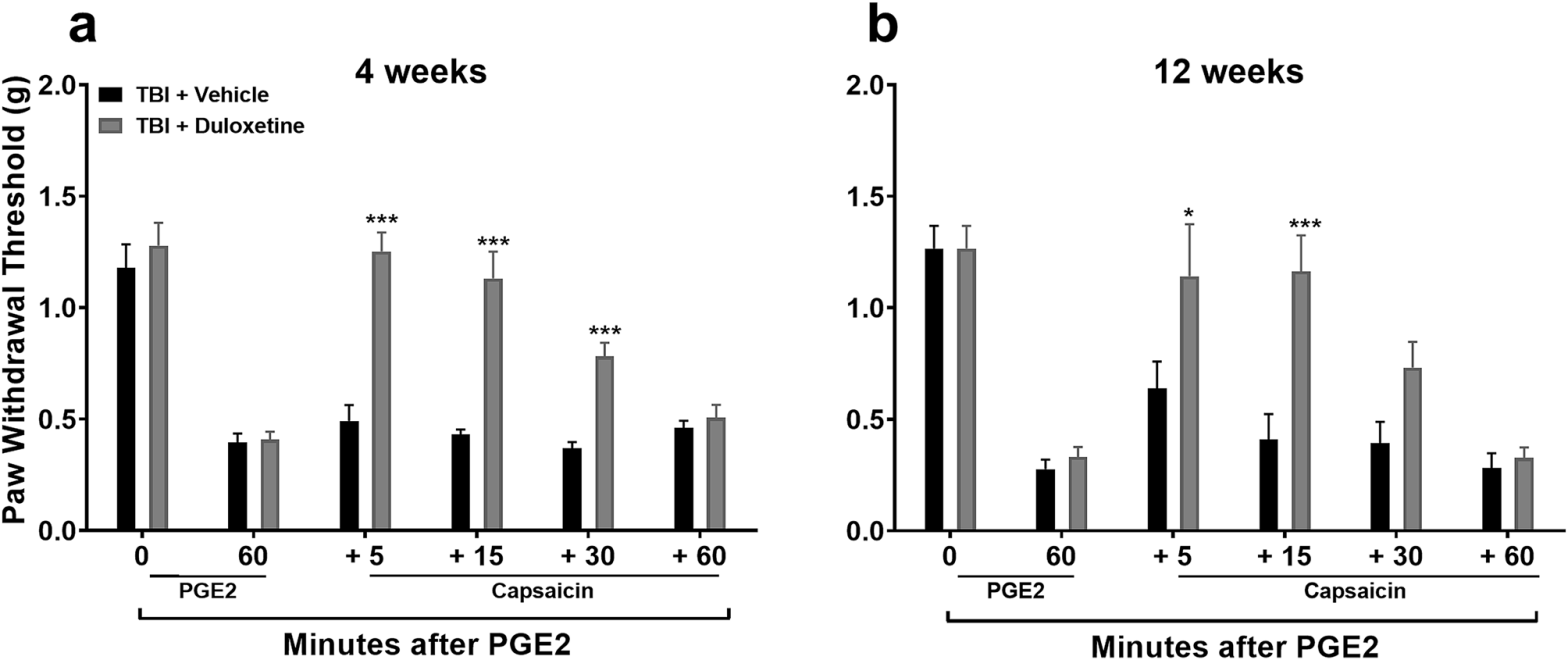
Assessment of diffuse noxious inhibitory control (DNIC) response after duloxetine (DUL) treatment. Systemic pre-treatment with DUL (10 mg/kg, s.c for 3 days), a serotonin and norepinephrine reuptake inhibitor, is effective in restoring the DNIC response at either 4 **(a**) or 12 (**b**) weeks post TBI in male mice. The last dose of DUL was given 18 h prior to testing to avoid its acute analgesic effects on PGE-2 application. Data were analyzed by two-way repeated measures ANOVA using the Sidak multiple comparisons test to correct for multiple comparisons. *p < 0.05, ***p < 0.001 indicates significant difference between vehicle and drug treatment groups. Error bars: SEM, n = 6/group.

Augmenting monoamine signaling by pretreating animals with reuptake inhibitors successfully restored DNIC responses in the rat model of TBI using lateral fluid percussion^16^. In this study we pretreated mice with the serotonin and norepinephrine reuptake inhibitor, duloxetine (DUL) prior to DNIC testing. The last dose was given 18 h prior to testing to avoid acute analgesic effects of DUL on PGE_2_ application. This mixed reuptake inhibitor was selected for testing because it is commonly used clinically for the treatment of other forms of chronic pain^25^. Systemic DUL effectively restored the DNIC response at both 4 (Fig. 7a: F_1,10_ = 54.13, *p* < 0.001; significant main effect of treatment) and 12 (Fig. 7b: F_1,10_ = 5.77, *p* < 0.05; significant main effect of treatment) weeks post TBI compared to vehicle treatment.

### Diffuse noxious inhibitory control (DNIC) response post-TBI was restored with selective serotonin reuptake inhibitor pretreatment

**Figure 8:**
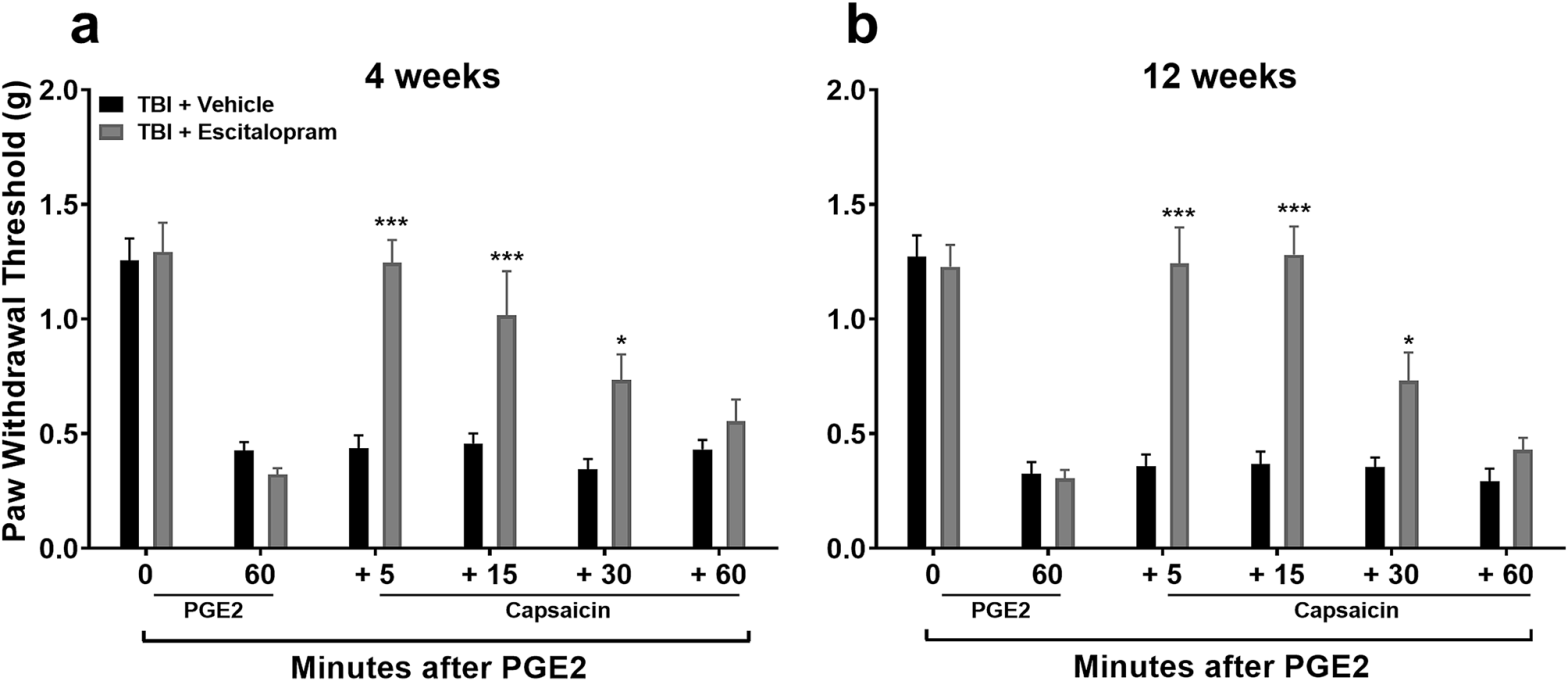
Assessment of diffuse noxious inhibitory control (DNIC) response after escitalopram treatment in TBI. Systemic pre-treatment with the serotonin-selective reuptake inhibitor, escitalopram (ESC, 10 mg/kg, i.p. for 3 days) does restore the DNIC response at either 4 (**a**) or 12 (**b**) weeks post TBI in male mice. The last dose of ESC was given 18 h prior to testing to avoid any potential acute analgesic effects of ESC on PGE_2_ application. Data were analyzed by two-way repeated measures ANOVA using the Sidak multiple comparisons test to correct for multiple comparisons. *p < 0.05, ***p < 0.001 indicates significant difference between vehicle and drug treatment groups.Error bars: SEM, n = 6/group.

Since NE-mediated contributions to DNIC seem to be limited after TBI in the mouse closed head TBI model, we went on to test a serotonin-selective reuptake inhibitor (SSRI) hypothesizing that it would be equally effective to an SNRI after TBI. Mice were pretreated with the SSRI escitalopram (ESC) for 3 days with the last dose given 18 h prior to testing to avoid potential acute analgesic effects of ESC on PGE_2_ application. This maneuver did restore the DNIC response at both 4 (Fig. 8a: F_1,10_ = 10.00, *p* < 0.01; significant main effect of treatment) and 12 (Fig. 8b: F_1,10_ = 47.75, *p* < 0.001; significant main effect of treatment) weeks post TBI. Escitalopram did not alter the DNIC response in control mice (Figure S3).

### Diffuse noxious inhibitory control (DNIC) response post-TBI was restored with selective serotonin reuptake inhibitor pretreatment in female mice

**Figure 9:**
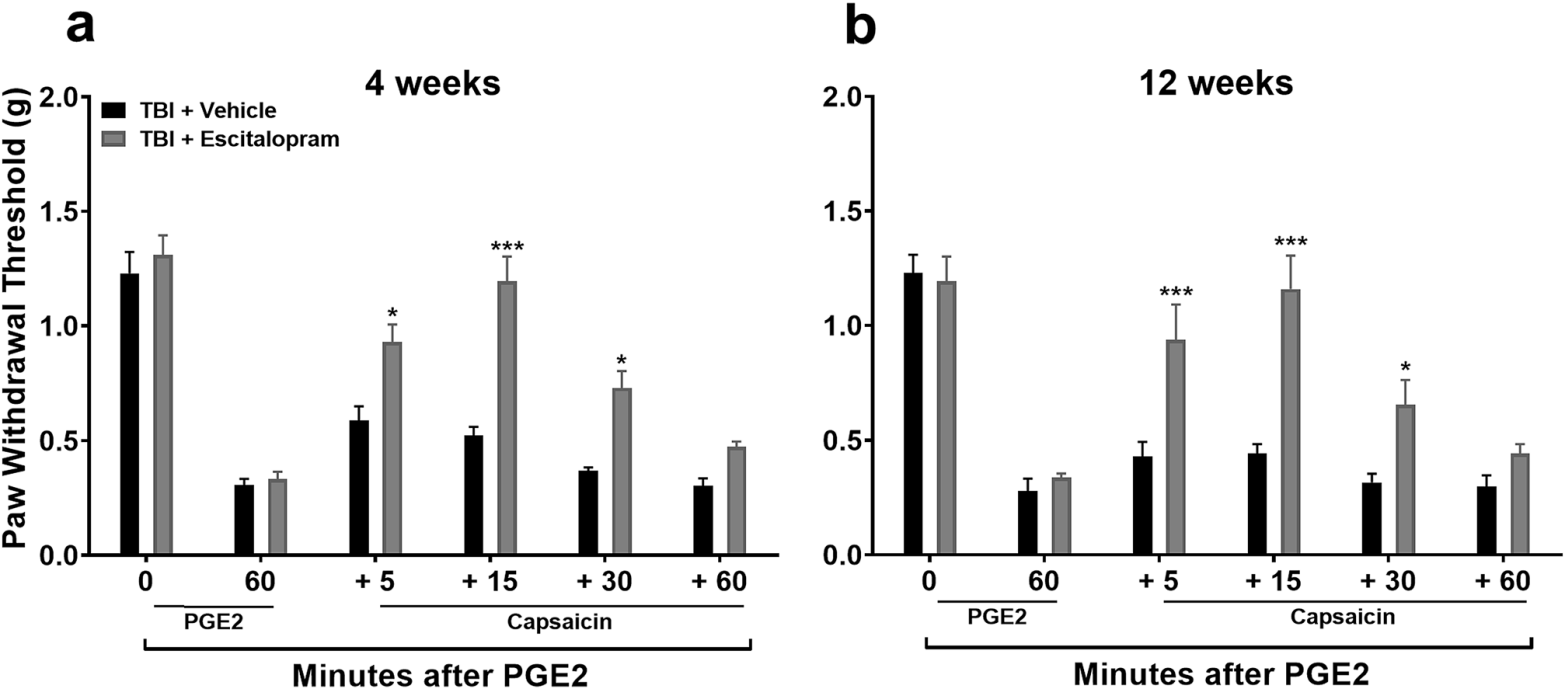
Effects of escitalopram treatment on diffuse noxious inhibitory control (DNIC) response after mild TBI in female mice. Systemic pre-treatment with the serotonin-selective reuptake inhibitor, escitalopram (ESC, 10 mg/kg, i.p. for 3 days) restores the DNIC response at either 4 (**a**) or 12 (**b**) weeks post TBI in female mice. The last dose of ESC was given 18 h prior to testing to avoid any potential acute analgesic effects of ESC on PGE_2_ application. Data were analyzed by two-way repeated measures ANOVA using the Sidak multiple comparisons test to correct for multiple comparisons. *p < 0.05, ***p < 0.001 indicates significant difference between vehicle and drug treatment groups. Error bars: SEM, n = 6/group.

Our data indicate that female mice show similar loss of DNIC comparable to males after closed-head TBI (Fig. 1), we therefore hypothesized that ESC would also work in female cohorts. Systemic pretreatment with ESC prior to DNIC testing at 4 (Fig. 9a: F_1,10_ = 27.85, *p* < 0.001; significant main effect of treatment) and 12 (Fig. 9b: F_1,10_ = 14.75, *p* < 0.01; significant main effect of treatment) weeks did, in fact, restore the DNIC response in TBI female mice. ESC did not alter the DNIC response in control female mice (Figure S3).

## Discussion

Traumatic brain injury (TBI) is a multifaceted injury that occurs by sudden trauma causing damage to the brain. A common consequence of TBI is chronic pain. Although the mechanistic explanation for this remains elusive, imbalance of descending pain modulatory pathways as an underlying relevant mechanism has been investigated^6,16,19,21,26^. Our previous work using experimental models of TBI has shown the loss of effective endogenous pain modulation, specifically diffuse noxious inhibitory control (DNIC) that occurs in the first month after TBI^19^. In the rat lateral fluid percussion injury, a model requiring the opening of the skull and a percussive wave applied to the dura, restoration of the DNIC response occurred after enhancement of descending antinociceptive, serotonergic signaling^21,27^. However, not all TBIs are the same; they can vary in severity from a mild or concussive injury to life threatening penetrating injuries. Concussion accounts for approximately 70–90% of patients treated for a TBI in the emergency room each year in the US^28^. It may be reasonable, then to extend early observations to a concussive model, explore the responsible mechanisms more thoroughly, and further expand the scope by reproducing key observations in both sexes.

In our lab, the mouse closed-head model of TBI was adopted to recapitulate certain features of the human concussive TBI injury^19,26,29,30^. Using this model, we have demonstrated that injured mice also manifest a two-stage pain process like that seen in the rat^19,26^. Initial acute onset (within 72 h) of hindlimb hyperalgesia lasting 14 days is followed by a loss of DNIC by 4 weeks post TBI, when recovery of peripheral mechanical hyperalgesia has occurred^19,26^. A key aim for this study was to investigate whether the mechanisms responsible for the loss of DNIC after a concussive injury in the mouse was comparable to the rat TBI injury^16,19,21^.

An important set of observations from the current work was that failure of the DNIC response after concussive TBI injury occurred in both male and female injured mice and lasted for at least 12 weeks post-injury, a timepoint consistent with most definitions of chronic pain in human populations. We then focused our investigations on assessing the role of the descending noradrenergic inhibitory pain pathway in the DNIC response in mice. Norepinephrine (NE) is released by descending fibers originating from the NE nuclei A5, A6 (locus coeruleus), and A7 in the brain stem targeting α2-adrenoceptors (α2-ARs) in the dorsal horn of the spinal cord. Treatment with the α2-AR antagonist atipamezole prior to the conditioning stimulus, capsaicin, blocked the manifestation of the DNIC response in naïve mice, confirming a central role for NE in descending pain inhibition under unperturbed states. However, treatment with NE reuptake inhibitor reboxetine to enhance spinal NE levels did not reinstate the DNIC response after mild TBI. One possibility for the lack of reboxetine effect to restore DNIC in TBI mice at both 4 and 12 weeks post injury could have been low spinal levels of NE after injury. However, quantification of NE in the lumbar spinal cord at 4 weeks post-injury revealed no change in TBI model mice compared to naïve mice. Alternatively, reboxetine pretreatment could have failed to reinstate the DNIC response due to a TBI-induced compromise in the function of the spinal α2-ARs. To test this, TBI and naïve mice were treated intrathecally with the α2-AR agonist, dexmedetomidine (DEX). This study revealed that responsiveness to the agonist DEX was in fact substantially reduced suggesting that spinal sensitivity to NE after TBI might account for some of the reduced DNIC response thus compromising endogenous pain control.

The next strategy to correct the imbalance in descending pain modulation and restore DNIC in TBI mice was to reduce descending facilitation by decreasing pronociceptive serotonergic (5-HT) signaling. Pronociceptive signaling has been found to be responsible for supporting early (first few weeks) hindpaw allodynia in TBI model animals^16^, while others have cited activation of the 5-HT_3_ receptor as a mechanisms for loss of DNIC in neuropathic pain models^24^. Quantification of lumbar spinal serotonin levels revealed a significant increase at post-injury compared to sham mice (Fig. 4) suggesting an increase in 5-HT expression and therefore pronociceptive signaling could be responsible for the DNIC failure in TBI mice. The 5-HT_3_ receptor is a ligand-gated ion channel which plays a substantial role in facilitating pain transmission in both the central and peripheral nociceptive systems. However, intrathecal treatment with the potent selective 5-HT_3_ receptor antagonist ondansetron prior to the conditioning stimulus failed to restore DNIC at 12 weeks post-injury.

The final strategy to restore DNIC after TBI was to augment descending serotonergic antinociceptive signaling in the spinal cord using clinically available serotonin reuptake inhibitors. Serotonergic fibers that descend to the dorsal horn from the rostral ventromedial medulla (RVM) can either inhibit or facilitate pain, depending on the receptor subtype activated. For example, serotonin binding to 5-HT_1A_, 5-HT_1B_, 5-HT_1D_ and 5-HT_7_ spinal cord receptors inhibits nociceptive signal transmission whereas the binding to 5-HT_3_ and also 5-HT_2A_ receptors causes enhances nociceptive signaling^31–34^. In this study, mice were pretreated with the SNRI, duloxetine (DUL), prior to DNIC testing to avoid the acute analgesic effect of the drug on PGE_2_ induced sensitization^21^. DUL-treated, TBI mice revealed a restoration of the DNIC response when compared to vehicle treatment. To more selectively modulate the serotonergic system, we pretreated mice with the clinically available drug escitalopram (ESC), a selective serotonin reuptake inhibitor. The DNIC response was restored in ESC-treated TBI male mice both at 4 and 12 weeks post-injury. Furthermore, the DNIC response was also reinstated in ESC-treated female mice at 4 and 12 weeks post-injury.

The results of these studies build on work reported by our laboratory and others regarding damage to endogenous pain control mechanisms caused by TBI. Clinical observations demonstrate reduced conditioned pain modulation (CPM, the DNIC analog in humans) amongst patients who have suffered a mild TBI^12^. This type of loss is consistent with data from human imaging studies suggesting damage to endogenous pain control centers caused by TBI^13^. Likewise, laboratory models examining neuroinflammation and axonal damage have provided evidence of such pathologies near the LC, RVM and other areas of the brain involved in sensory signaling and pain modulation^6,19^. The findings of the present study add robust evidence that damage to endogenous pain control processes after TBI is clearly demonstrable in both rat and mouse models of mild TBI, and that both sexes appear to be affected similarly. Importantly, augmenting serotonergic signaling as opposed to noradrenergic signaling more commonly used in targeting neuropathic, musculoskeletal and other forms of chronic pain, may be more effective. The clinically available medications duloxetine and escitalopram were both highly efficacious in our mouse and rat models, and do have translational value for making clinical trials justifiable. An intriguing possibility, although untested in these studies, is that SSRI medications could augment the depression, anxiety, and memory problems commonly experienced after TBI. The approved indications for SSRIs already include anxiety and depression. It should be kept in mind, however, that distinct mechanisms may support these other complaints^35–37^.

The present studies have important limitations, however. One of the most important is that we studied a pain control mechanism strongly linked to human pain states^38^, but we did not model a specific type of pain in the animals. Thus, looking at the effects of SSRI’s in animals with TBI in headache, limb pain, joint pain or other models may be helpful. Also, the majority of our testing used reflexive endpoints rather than more complex functional or behavioral ones that might provide better insight into the consequences of disrupted DNIC. Lastly, while rodent models are convenient, a larger animal model may provide a useful intermediate testing platform on which to evaluate the effects of drugs like SSRIs in TBI models. In any case, pain after TBI has no specific set of available treatments, and there is significant clinical need to advance this avenue of research.

## Materials and methods

### Animals

All experiments were done with male or female C57Bl/6 J mice (Jackson Laboratories; Bar Harbor, MA, U.S.A). Upon arrival at our animal facility, mice were acclimated for at least 7 days before beginning of experimentation. The mice were 12 weeks old at time of experiments and were kept under standard vivarium conditions of 12 h light/dark cycle, ambient temperature and were given food and water ad libitum. Mice were habituated to handling by the experimenters. The Veterans Affairs Palo Alto Health Care System Institutional Animal Care and Use Committee (Palo Alto, CA, USA) approved all experimental procedures and protocols in accordance with the guidelines of National Institutes of Health Guide for the Care and Use of Laboratory Animals. In addition, the study is reported in accordance with ARRIVE guidelines. Key findings from experiments done with male mice were later replicated with female mice to evaluate potential sex differences in outcomes. Experimenters were blind to the identity of treatments or experimental conditions. Based on preliminary data and our prior publications group sizes determined for each outcome by power analysis for 30% differences detectable with 80% power at alpha 0.05 were 6–8 per group.

### Closed-head model of mild traumatic brain injury (mTBI)

The closed head mTBI and sham procedures were based on our previously established protocols^19^. Briefly, a benchmark stereotaxic impactor (MyNeurolab, St. Louis, MO, USA) actuator was mounted on a stereotaxic frame (David Kopf Instruments, Tujunga, CA, USA) at a 40° angle with a 5 mm impactor tip. After isoflurane anesthesia induction, mice were placed in a foam mold, held in prone position on the stereotaxic frame, and maintained under anesthesia for the duration of the procedure. The stereotaxic arm was adjusted so that the head impact was at a fixed point relative to the right eye and ear, corresponding to the S1 somatosensory cortex. The impact delivered by the device to the head was 5.8–6.0 m/s with a dwell time of 0.2 s and impact depth of 5 mm. The above parameters result in a mild TBI **(**Figure S1**)** as measured by mouse neurological severity score (NSS), evaluated 1 h post injury. After impact, the mice recovered from anesthesia on a warming pad prior to returning to their home cages. For sham control groups, the above procedure was performed except that the impact device was discharged in the air.

### Neurological severity score (NSS)

The neurological status of TBI mice was evaluated at different time intervals post injury using the NSS scale. The neurobehavioral and motor assessments of NSS evaluates performance in 10 different tasks. The mice are evaluated for motor ability, balance and level of alertness. One point is given for failing to perform each task and a score of 0 for the ability to perform it. Therefore, an uninjured mouse scores 0 and the maximum score is 10. NSS evaluation at 1 h post injury is considered to be an important indicator of the severity of injury. NSS less than 5 points is considered to be mild injury, moderate injury with NSS of 5–6, severe injury with an NSS of 7–8, and very severe injury would be an NSS of 9–10 in mice^39,40^.

### Mechanical nociceptive assay

Mechanical sensitivity was assessed as described previously^41^ using nylon von Frey filaments (Stoelting Co., IL, USA) according to the “up-down” algorithm developed by Chaplan et al^42^. We have applied this technique previously to estimate withdrawal thresholds in mice after TBI^19,26^. After acclimating mice on the wire mesh platform inside plastic enclosures (10 cm radius), sequential fibers with increasing stiffness were applied to the plantar surface of hind limb and left in place for 5 s. When 4 fibers had been applied after the first response the testing was terminated. Withdrawal of hind paw from the fiber was considered a response. If a response occurred after application of a fiber then a less stiff fiber was applied, if no response was observed the next stiffest fiber was applied. Mechanical withdrawal threshold was determined by a data fitting algorithm to allow for determination of significance using parametric statistical analysis^43^.

### Diffuse noxious inhibitory control (DNIC) assessment

DNIC assessment of post trauma mechanical hypersensitivity was done using a noxious stimulation–induced analgesia protocol modified for mice^44,45^. DNIC assessments in the TBI groups were done after mice had recovered to baseline mechanical threshold levels (4 or 12 weeks). Initially, left hind paw Prostaglandin E2 (PGE_2_) injections were given to produce brief hypersensitivity. Next, mice were tested after 1 h for hindpaw mechanical hypersensitivity and subsequently got an ipsilateral forepaw capsaicin injection. The amount of DNIC induced by capsaicin injection was evaluated by hind paw mechanical threshold testing.

### Drug treatments

In order to assess DNIC in TBI and control groups, hind paw injection of PGE_2_ (100 ng/15 µl s.c., Cayman Chemicals, USA) preceded forepaw capsaicin (7.5 µg/5 µl s.c.; Sigma-Aldrich, USA) application. Stock PGE_2_ solutions were made in 100% ethanol and further diluted (1:1000) in 0.9% sterile saline prior to use. Capsaicin was dissolved in sterile saline solutions containing 0.25% DMSO, 0.25% ethanol and 0.125% Tween 80 and prepared daily prior to use. For systemic α2 adrenoceptor blockade, mice were treated with atipamezole (ATZ; 1 mg/kg, i.p., Sigma-Aldrich, MO, USA) once after mechanical threshold testing for PGE_2_ sensitization was completed. A similar timeline was used for dexmedetomidine (100 µg/5 µl, i.th., Cayman Chemicals, USA) testing. To assess the effect of the selective serotonin 5-HT_3_ receptor blockade on DNIC response in TBI and control groups, ondansetron (OND; 1 µg/5 µl, i.th., Sigma-Aldrich, USA) was used. To assess the relative contribution of serotonin (5-HT) and norepinephrine (NE) signaling in DNIC deficits after TBI, selective 5-HT and NE reuptake inhibitors were employed. Mice were treated with reboxetine (RBX; 10 mg/kg, i.p., Thermo Fisher, MA, USA), escitalopram (ESC; 10 mg/kg, i.p., Thermo Fisher, MA, USA) or duloxetine (DUL; 10 mg/kg, s.c., Thermo Fisher, MA, USA) for 3 days prior to testing. The above drugs were diluted in sterile saline immediately prior to use. Intrathecal injections were done after brief isoflurane anesthesia with a 30G needle between the groove of L5 and L6 vertebrae. Successful entry of the needle into the intradural space was confirmed by the observation of a tail flick. Following the injection, mice were recovered using standard procedure.

### Enzyme immunoassay

Mouse spinal cord (L4, 5 lumbar enlargement) was collected and frozen immediately on dry ice. The spinal cord tissue was cut into fine pieces in ice-cold PBS, pH 7.4, containing a cocktail of protease inhibitors (Roche Applied Science, NJ, USA) followed by homogenization using TissueLyser LT (QIAGEN, MD, USA). Homogenates were centrifuged for 15 min at 12,000 g, 4 °C. The supernatants were aliquoted and stored at − 80 °C until required for enzyme immunoassay (EIA) performance. Total protein contents in all tissue extracts were measured using the DC protein assay kit (Bio-Rad, CA, USA). Mouse serotonin and norepinephrine levels were determined in duplicate using respective EIA kits (MyBioSource, CA, USA), according to the manufacturer’s instructions. These assay systems detect mouse serotonin and norepinephrine with sensitivity of 0.26 ng/ml and 5 pg/ml, respectively. The concentrations of serotonin and norepinephrine proteins were calculated from the standard curve at each assay. Each protein concentration was expressed as ng/mg or pg/mg total protein.

### Data analysis

Mechanical sensitivity data were analyzed by by two-way repeated measures ANOVA using the Sidak method to correct for multiple comparisons. Two tailed t tests were used for EIA protein data analysis comparisons. Data for the above are presented as mean ± S.E.M for all analyses and significance determined as *p* < 0.05. NSS data were analyzes by Mann–Whitney test using Holm-Sidak method for multiple comparisons and presented as median with interquantile range.

## Supplementary Information

Supplementary Information 1.

Supplementary Information 2.

Supplementary Information 3.

## Data Availability

The datasets generated during and/or analyzed during the current study are available from the corresponding author on reasonable request.
