# Supplementary figures and images for: Monoamine control of descending pain modulation after mild traumatic brain injury
Source: Sci Rep. 2022 Sep 29;12:16359. doi: 10.1038/s41598-022-20292-7 (PMC9522857; doi:10.1038/s41598-022-20292-7)

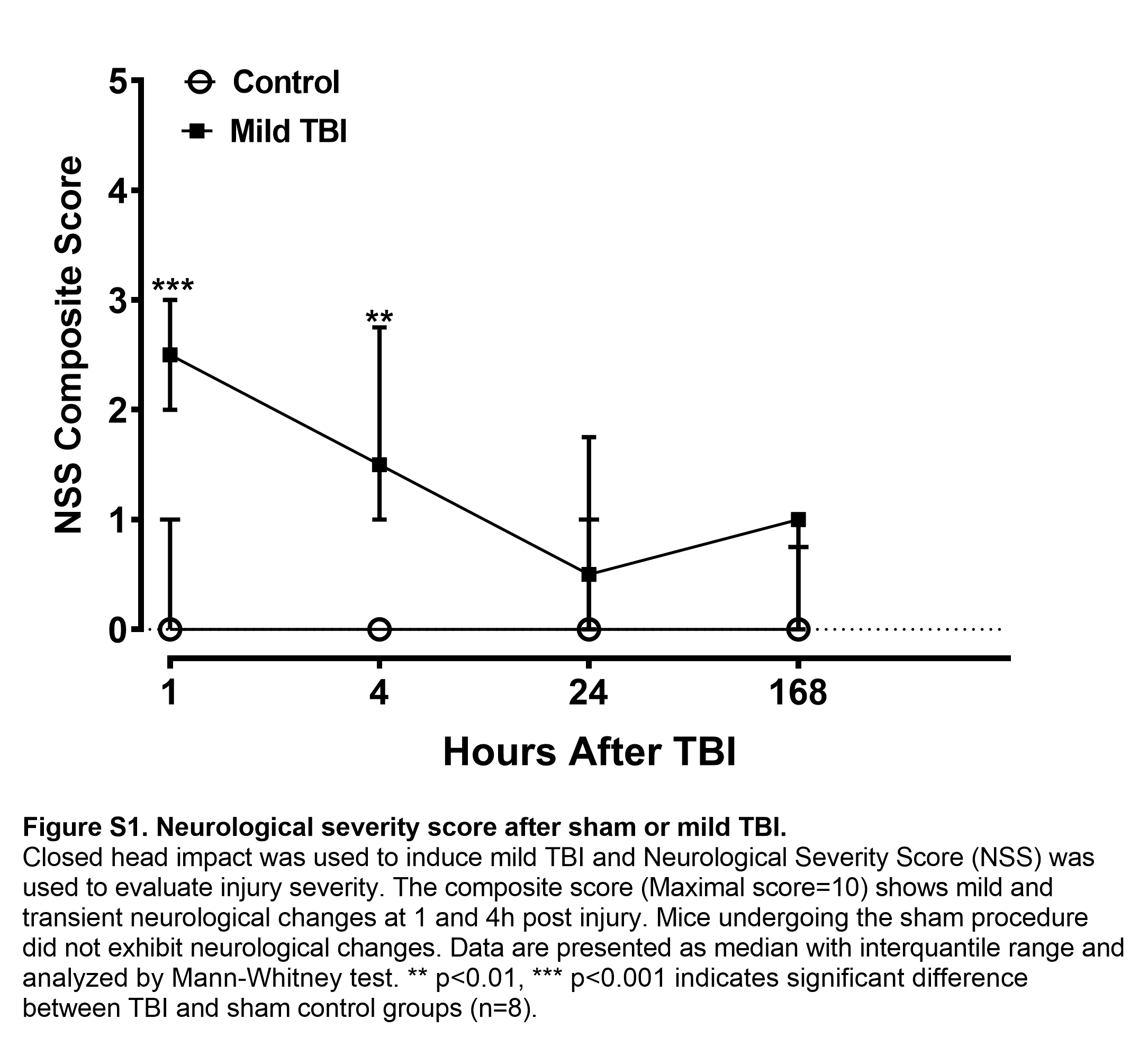

Supplement: Supplementary file 1 — Supplementary Information 1. [file 41598_2022_20292_MOESM1_ESM.tif]

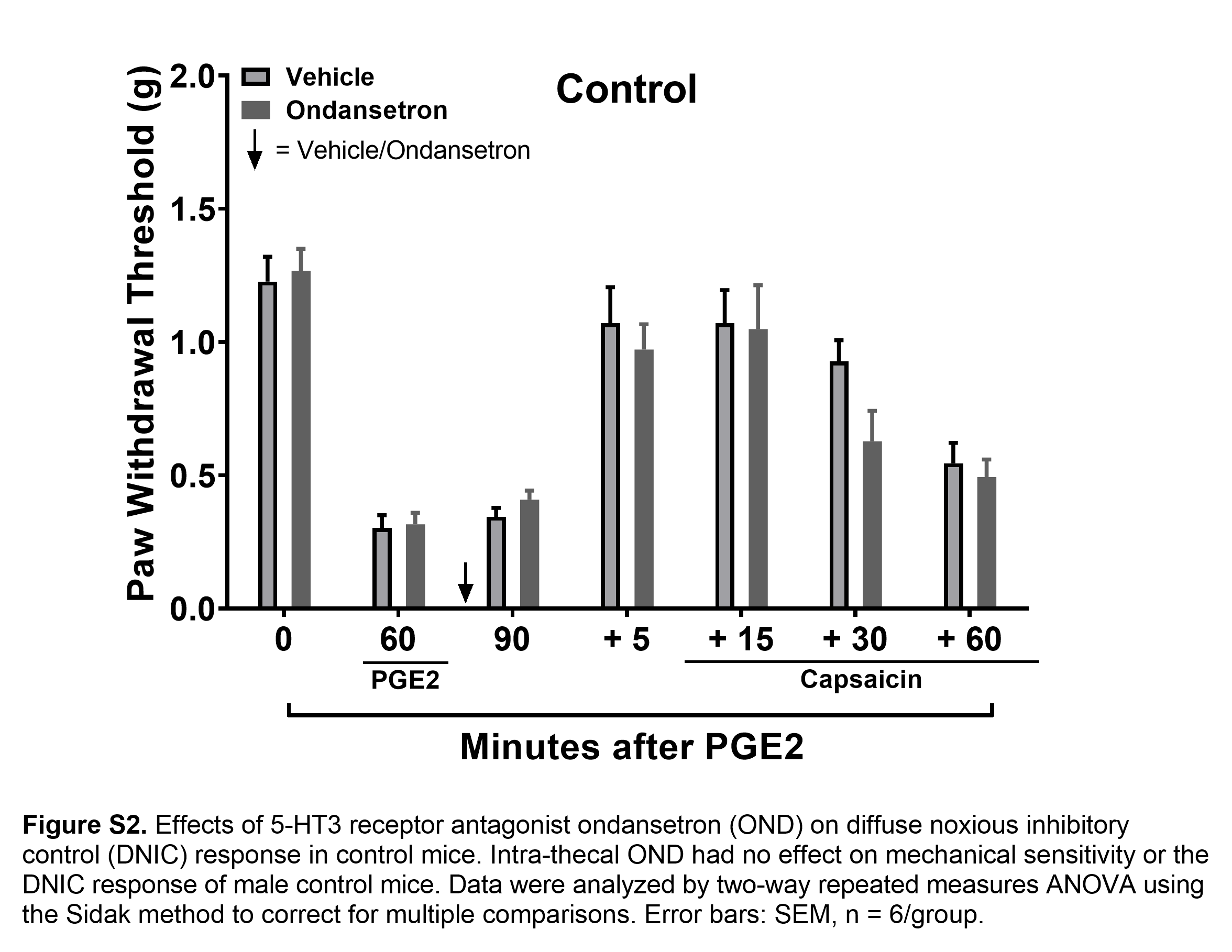

Supplement: Supplementary file 2 — Supplementary Information 2. [file 41598_2022_20292_MOESM2_ESM.tif]

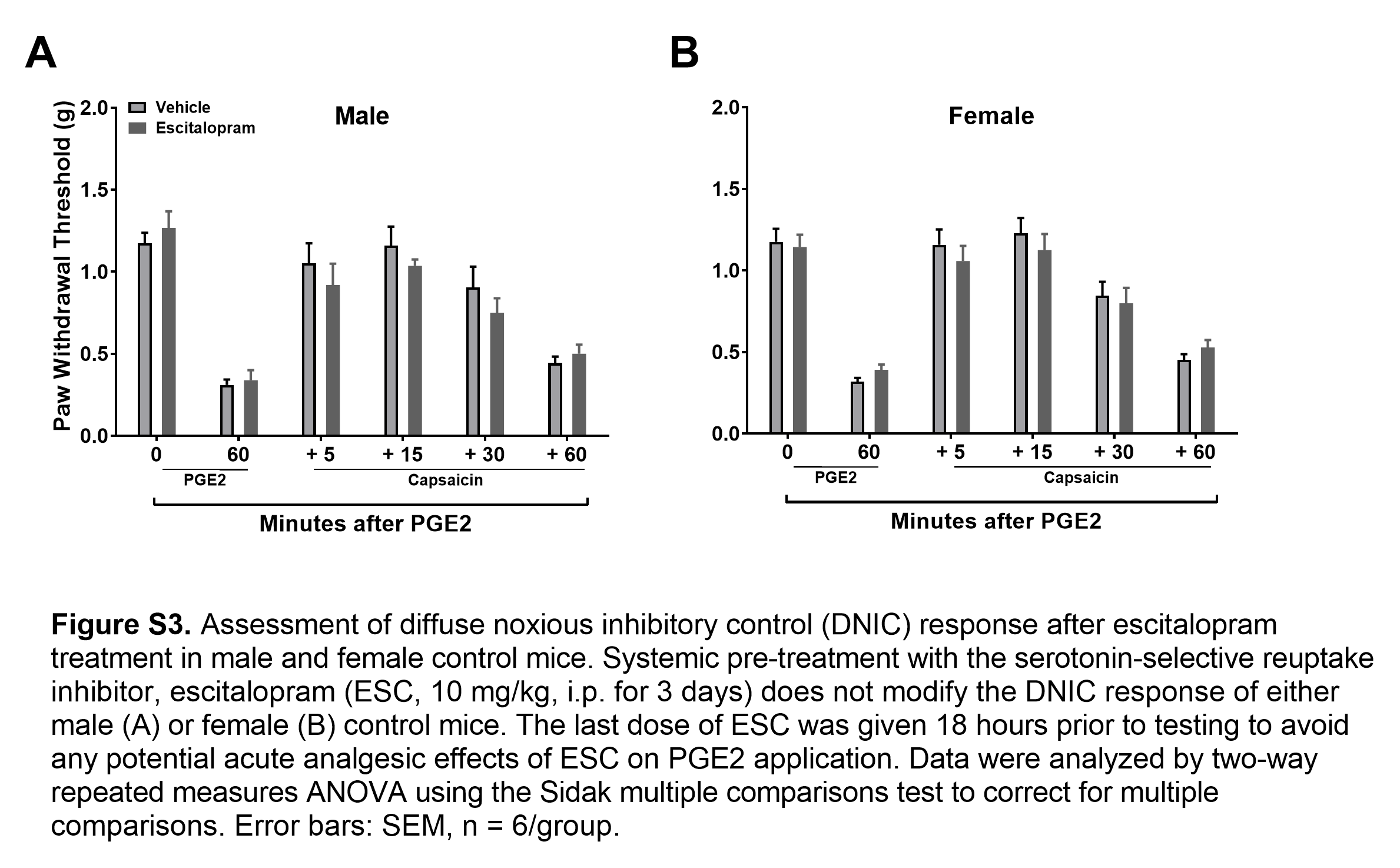

Supplement: Supplementary file 3 — Supplementary Information 3. [file 41598_2022_20292_MOESM3_ESM.tif]
